# Long-term crop residue application maintains oil palm yield and temporal stability of production

**DOI:** 10.1007/s13593-017-0439-5

**Published:** 2017-07-28

**Authors:** Hsiao-Hang Tao, Jake L. Snaddon, Eleanor M. Slade, Jean-Pierre Caliman, Rudi H. Widodo, Suhardi, Kathrine J. Willis

**Affiliations:** 1 Department of Zoology, University of Oxford, Oxford, Oxfordshire UK; 2 Centre for Biological Sciences, University of Southampton, Southampton, UK; 3 Lancaster Environment Centre, Lancaster University, Lancaster, Lancashire UK; 4 SMART Research Institute, Pt SMART, Pekanbaru, Riau Indonesia; 5 Royal Botanical Gardens, Kew, Richmond, Surrey, UK; 6 Department of Biology, University of Bergen, Bergen, Norway

**Keywords:** *Elaeis guineensis*, Crop residue, Yield stability, Empty fruit bunch, Perennial crop, Soil organic carbon, Chemical fertilizer, Sustainable oil palm, Relative humidity, Indonesia

## Abstract

Crop residue management is an important agricultural practice that has a high potential to improve soil health and optimize crop production. Compared to annual crops, relatively little is known about crop residue management effects on the yield and temporal stability of perennial crop production. This study focused on oil palm (*Elaeis guineensis*), an important tropical crop that had expanded rapidly over the past decades. We aimed to understand the effects of applying a major oil palm residue, the empty fruit bunch, on crop yield and temporal stability of production. We compared 15 years of crop yield performance from a field trial in Sumatra, Indonesia. The treatments included empty fruit bunch application of three application rates (30, 60, and 90 t ha^−1^ year^−1^), and a reference treatment of chemical fertilizers with no addition of empty fruit bunch. Compared to the reference treatment, the cumulative crop yield over 15 years under low, medium, and high application rates of empty fruit bunch increased by 2.4, 5.9, and 4.8%, respectively. The annual crop yield and temporal stability in production were not significantly different between treatments. Soil organic carbon was significantly higher under medium application rate of empty fruit bunch compared to that under the chemical fertilizer treatment. Soil organic carbon and relative humidity were positively associated with annual crop yield with a time lag of 2 years. This study is the first to show that both crop yield and temporal variability of oil palm production can be maintained under crop residue application, compared to chemical fertilizer treatment. Furthermore, climatic conditions had strong effects on the temporal variability of oil palm production. These findings will inform the design of optimal empty fruit bunch application schemes that enhance sustainable intensification of oil palm cultivation.

## Introduction

Optimizing agricultural management practices to enhance ecosystem health and maintain high crop yield is important for the sustainable development of agriculture (Garnett et al. 2013). Crop residue application is a widely used management practice which can benefit soil fertility and ecosystem functioning by providing trophic resources to the soil, modifying the soil abiotic environment, and enhancing soil biological activities (Edmeades 2003). Optimizing crop residue management is especially important for oil palm (*Elaeis guineensis*), an economically important tropical crop which produces vegetable oil widely used in the production of food and detergents and as a feedstock for biofuel. The land area under oil palm cultivation has reached 16.4 million ha globally in 2014, equivalent to 10% of the world’s permanent croplands (FAO 2015; Kurnia et al. 2016). More than half of the world’s plantations are located in Malaysia and Indonesia; these two countries produced 85% of the 56 million tons of crude palm oil produced worldwide in 2013 (FAO 2015). The cultivation of oil palm can lead to soil degradation, through the removal of understory vegetation, intensive use of chemical fertilizers, and the lack of carbon returns from crop residues (Guillaume et al. 2015). In the past decade, the practice of applying oil palm residues with reduced chemical fertilizers has increased within oil palm plantations, to replace the conventional practice using chemical fertilizers as the sole nutrient inputs (Singh et al. 2010). Crop residue application in oil palm has been shown to positively influence soil quality and soil ecosystem functions (Comte et al. 2013; Tao et al. 2016). However, the effects of crop residue application on oil palm yield remain unclear (Abu Bakar et al. 2010; Chiew and Rahman 2002). The uncertainty of yield responses under crop residue application and the lack of identification of optimal application schemes remain obstacles for the informed use of this practice (Fairhurst and Griffiths 2014).

The effects of crop residue addition on crop production are highly associated with climatic conditions and soil characteristics (Edmeades 2003; Rusinamhodzi et al. 2011). Crop residue application influences crop yield through different mechanisms; one of which is through increasing soil organic carbon (Lal 2010). Climatic factors can affect the decomposition rate of crop residues, which in turn influence soil organic carbon and crop yield (Rusinamhodzi et al. 2011; Ventrella et al. 2016). The potential temporal fluctuations in crop residue decomposition can therefore result in pronounced temporal variations in available soil nutrients, soil carbon, and thus crop production. In comparison, chemical fertilizers may serve as a more stable and readily available mineral nutrient source, contributing to a more stabilized crop yield over time. When the cumulative crop productivity is similar, farms with higher temporal stability in yield are likely to have better operations and economic returns (Fairhurst and Griffiths 2014). Climatic conditions such as radiation and water supply also directly influence crop yield, and these effects may override the effects of management practices (Rusinamhodzi et al. 2011). The majority of current studies have focused on the net changes in annual crop yield under crop residue addition; relatively little information is available on temporal changes in perennial crop yield under crop residue management, especially in tropical regions (Edmeades 2003).

**Fig. 1:**
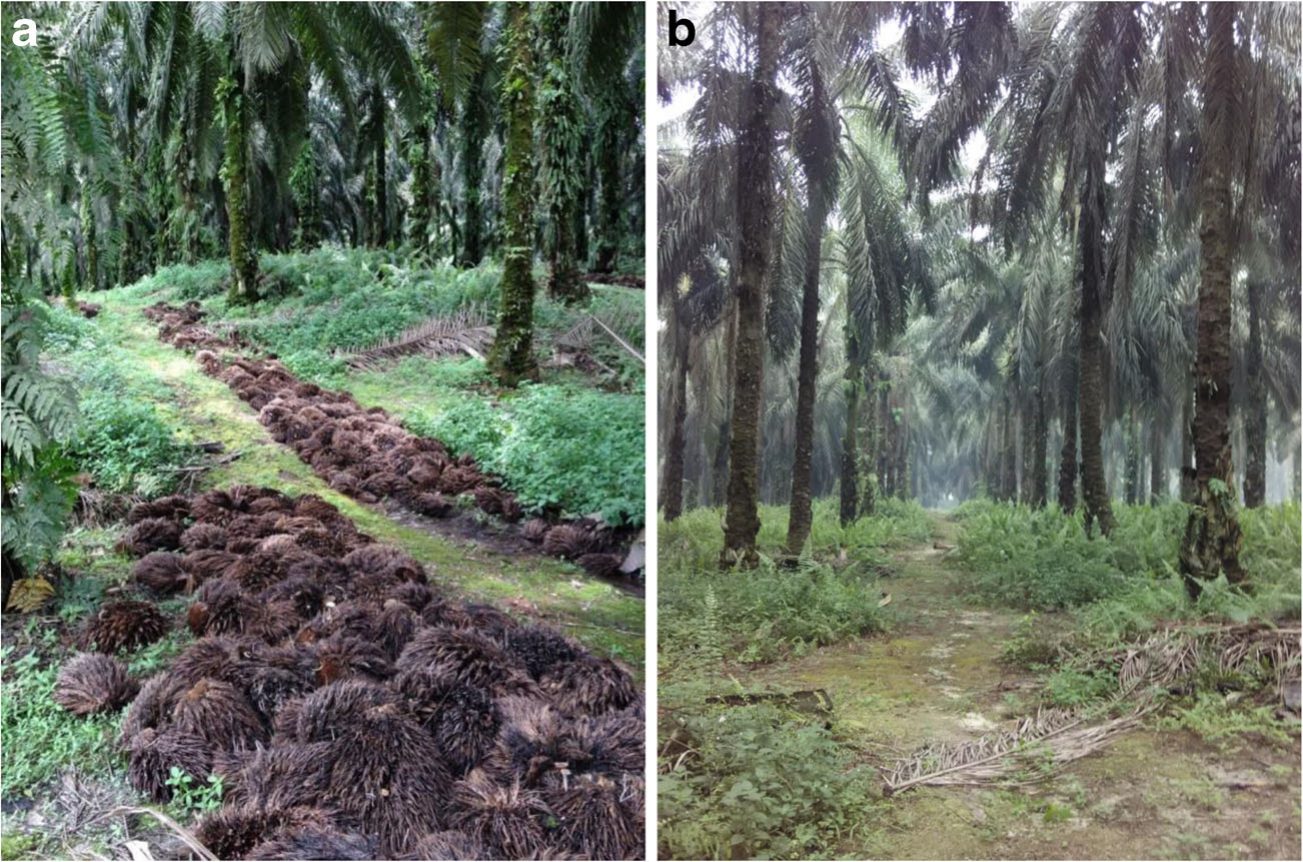
**a** An empty fruit bunch (EFB) treatment plot at the field trial in an oil palm plantation in Sumatra, Indonesia. The EFB was applied at the sides of the harvesting paths, and urea was applied on the top of EFB layers to facilitate EFB decomposition. **b** A reference treatment plot at the field trial. Chemical fertilizers were applied in the palm circles, without the application of EFB

The aim of this study was to examine the effects of a major oil palm residue, empty fruit bunch (EFB), on oil palm yield and temporal stability of production. EFB is the structural part of fruit bunches after fruit removal for the oil extraction. A plantation with fresh fruit yielding of 30 t ha^-1^ year^-1^ generates approximately 6.6 t ha^-1^ year^-1^ of EFB (Yusoff 2006). For oil palm mills close to plantations, EFB is often returned to plantations as mulch substrate to improve soil quality. However, due to the high cost of transportation and application, EFB is often used alternatively as feedstock for bioenergy or raw material for composts (Wiloso et al. 2015). In this study, we investigated crop yield performance over 15 years of continuous EFB application with three application rates (30, 60, and 90 t ha^−1^ year^−1^ for low, medium, and high rates, respectively) and a reference treatment as control, in a field trial in an oil palm plantation in central Sumatra, Indonesia (Fig. 1). The application rates of low- and medium-EFB treatments were within the range of 15 to 60 t ha^−1^ year^−1^ of standard practices in the oil palm industry (Pardon et al. 2016). Alternatively, the high-EFB treatment represented higher organic matter inputs than common practices. The reference treatment followed a standard estate practice of chemical fertilizers without the addition of EFB. The potential effects of climatic conditions and soil properties on crop yield were also examined. We asked (H1) whether different treatments and climatic factors influenced crop yield over time, (H2) whether different treatments affected the temporal stability in crop yield, and (H3) whether the effects of treatment on crop yield were associated with soil organic carbon levels. We hypothesized that compared to the reference treatment, EFB application would either maintain or increase crop yield, and the magnitude of effects would depend on the application rate of EFB. We further hypothesized that climatic conditions would pose strong effects on crop yield, and that the yield stability over time would be reduced under EFB application. Finally, we expected that EFB application would enhance crop yield by increasing soil organic carbon.

## Materials and methods

### Site description

The study was carried out in an oil palm plantation in Riau Province of Central Sumatra, Indonesia. The oil palm plantation was the first generation established in 1987. It is certified by the Roundtable on Sustainable Palm Oil (RSPO). The previous land use of this area was tropical lowland secondary forest dominated by *Dipterocarp* species. The climate of this region is described as tropical humid, with a mean temperature of 26.8 °C and average rainfall of 2400 mm year^−1^ (230 mm month^−1^ in the wet season and 140 mm month^−1^ in the dry season). The soils are Inceptisols of Typic Dystrudepts (USDA soil classification system), with the loamy lowland soil class.

### Experimental design

The 15-year trial began in 1998, when the age of oil palms was 11 years. The field trial was established in two adjacent management blocks, in a flat area with limited leaching and runoff. The field trial was composed of five replicate blocks, covering a total area of 36 ha of 1200-m length and 300-m width. Each of the five replicate blocks had four treatment plots: low-EFB treatment (30 t ha^−1^ year^−1^, equivalent to 210 kg palm^−1^ year^−1^), medium-EFB treatment (60 t ha^−1^ year^−1^, equivalent to 420 kg palm^−1^ year^−1^), high-EFB treatment (90 t ha^−1^ year^−1^, equivalent to 630 kg palm^−1^ year^−1^), and a reference treatment of chemical fertilizers with no EFB application.

Each treatment plot was surrounded by 1.5-m ditches to minimize interference from adjacent treatment plots. Each treatment plot was composed of 36 palms located in 4 rows, with a plot size of approximately 80-m length and 40-m width. The 12 oil palms in the centre of each treatment plot were used as focal palms for crop productivity and soil property measurements. In the EFB treatment plots, EFB was applied once a year at one side of the harvesting paths, followed by urea application on the top of EFB to accelerate the EFB decomposition. In the reference treatment plots, chemical fertilizers were applied within palm circles twice a year (i.e. during the February–March and September–October periods) throughout the trial period. The application rate, frequency, application location, and type of chemical fertilizers for each treatment are detailed in Table 1.

**Table 1:** The application rates of empty fruit bunch (EFB) and chemical fertilizers, and the equivalent nutrient application rates for the reference treatment, low-EFB treatment, medium-EFB treatment, and high-EFB treatment

Code	Treatment	Application rate (kg palm^−1^ year^−1^)	Nutrient application rate (kg palm^−1^ year^−1^)						Cumulative fresh fruit bunch weight over 15 years from 5 replicate plots (t ha^-1^) with percent increase compared to reference
			C	N	P	K	Mg	Ca	
Reference	Chemical fertilizers without the addition of EFB	Urea 1.75		0.81					2040
		TSP 0.5			0.13			0.07	
		MOP 2.5			0.38	1.25			
		Kieserite 0.05					0.08		
Low-EFB	Low application rate of EFB (30 t ha^−1^ year^−1^) with urea	EFB 210	102	0.56	0.064	1.7	0.1	0.1	2088 (+2.4%)
		Urea 0.02		0.01					
Medium-EFB	Medium application rate of EFB (60 t ha^−1^ year^−1^) with urea	EFB 420	204	1.12	0.13	3.4	0.2	0.2	2161 (+2.9%)
		Urea 0.04		0.02					
High-EFB	High application rate of EFB (90 t ha^−1^ year^−1^) with urea	EFB 630	306	1.68	0.19	5.1	0.3	0.3	2137 (+4.8%)
		Urea 0.06		0.03					

The carbon and nutrient composition of EFB was referenced from Comte et al. 2013; Moradi et al. 2014

*EFB* empty fruit bunch, *TSP* triple super phosphate (Ca(H_2_PO_4_)_2_·H_2_O), *MOP* muriate of potash, potassium chloride (KCl), *Kieserite* magnesium sulfate (MgSO_4_·H_2_O)

### Measurements of oil palm yield and soil properties

The fresh fruit bunch weight was used as an indicator for oil palm yield in our study, as there is a fixed ratio between the fresh fruit bunch weight and extracted palm oil for the same variety of oil palm (Squire 1986). The fresh fruit bunches from the 12 focal palms at each treatment plot were harvested and weighed throughout the trial. The 15-year cumulative yield was the sum of annual yield from five replicate plots of the same treatment.

Soil samples at 0–15-cm depth were collected at palm age of 13, 16, 19, 23, and 26 years (equivalent to 2, 5, 8, 12, and 15 years of application). Soils were taken at the positions beneath the EFB (at the side of harvesting paths) in the EFB treatment plots, and at the equivalent positions in the reference treatment plots, in order to examine the localized effects of EFB on soil organic carbon. As chemical fertilizers applied in oil palm plantations have limited spill-over effects (Carron et al. 2016), we assumed that the chemical fertilizers applied within the palm circles in the reference treatment plots have limited influences on soil properties at the nearby harvesting paths. Soils collected from 12 focal palms of each treatment plot at each time point were pooled to determine the soil organic carbon concentration, using the Walkley-Black method (Nelson and Sommers 1982). The climatic variables including annual values of maximum temperature, minimum temperature, mean temperature, rainfall, and relative humidity were measured at a meteorological station approximately 5 km from the trial site throughout the study.

### Statistical analysis

We used linear mixed effects models for statistical analyses in R 3.2.2 with the *lme4* package (R Core Team 2016). Three hypotheses were tested: first, whether EFB application and climatic factors influenced crop yield over the application period (H1); second, whether EFB application affected the temporal stability in crop yield (H2); and third, whether EFB application influenced crop yield by altering soil organic carbon levels (H3).

Prior explorations of climatic factors (annual rainfall, air temperature, and relative humidity) were conducted for their potential effects on yield. We examined the effects with 1 or 2 years of time lag, as climate conditions may have delayed effects on oil palm yield (Corley and Tinker 2015). The pre-test showed that relative humidity was the only climatic factor as a driver factor for crop yield, with lagged effects of 2 years. We thus included relative humidity as a covariate in H1. The fixed effects also included the interaction terms of treatment type with application year and with the quadratic and cubic terms of application year. This was to capture the temporal dynamics of crop production. The resulting fixed effects structure of the initial model in the R syntax was as follows: ~ treatment × year + treatment × year^2^ + treatment × year^3^ + humidity. The replicate block was included as a random effect to account for the spatial correlations of treatment plots within the same block. The application year was also included as a random effect to be accounted for temporal correlations of the repeated-measured data. We used stepwise deletion by the ANOVA function to drop non-significant variables (*P* > 0.05), before comparing the most parsimonious model with the null model (Zuur et al. 2009). The Tukey HSD analysis was proceeded as the post hoc test when the overall difference between the treatment types was observed.

We then examined the treatment effects on yield stability over 15 years (H2). We followed the calculation of temporal stability in the discipline of ecosystem ecology, with the concept that higher yield stability represents a lower inter-annual yield variability (Hautier et al. 2014). The temporal stability of crop yield for each treatment plot was defined as *μ*/*σ*, where *μ* is the temporal mean of crop yield and *σ* is the temporal standard deviation of crop yield over 15 years. For each treatment plot, *μ* was calculated as the mean of annual crop yield over 15 years, and *σ* was the standard deviation of annual crop yield over 15 years.

Lastly, we tested whether the effects of treatment on crop yield were positively associated with soil organic carbon levels (H3), by testing if the treatment type had an effect on soil organic carbon (H3.1), and if soil organic carbon had an effect on crop yield (H3.2). Before testing H3.1, we explored the potential role of climatic factors on soil organic carbon, because climate factors may either affect crop yield by providing favourable conditions for palm growth, or by altering soil organic carbon levels i.e. by affecting litter decomposition and nutrient release with potential time lags (Couteaux et al. 1995). Our pre-test showed that none of the climatic factors significantly explained soil organic carbon. This result suggested that climatic factors directly affected crop yield, mainly through sufficient water supply that facilitated nutrient uptake and palm growth. Therefore, we did not include climatic factors in the H3.1 model but in the H3.2 model as a covariate. To test H3.1, we included the interaction term of treatment type and application year as fixed effects, specified as ~ treatment × year in the R syntax. For testing H3.2, we included soil organic carbon and relative humidity as fixed effects, specified as ~ soil organic carbon + humidity in the R syntax. We examined the lagged effects of soil organic carbon for 1 and 2 years, because soil properties can have delayed effects on oil palm growth (Fairhurst and Griffiths 2014). For both H3.1 and H3.2 models, the replicate block and application year were included as the random effects.

## Results and discussion

### EFB application effects on crop yield and temporal stability of production

**Fig. 2:**
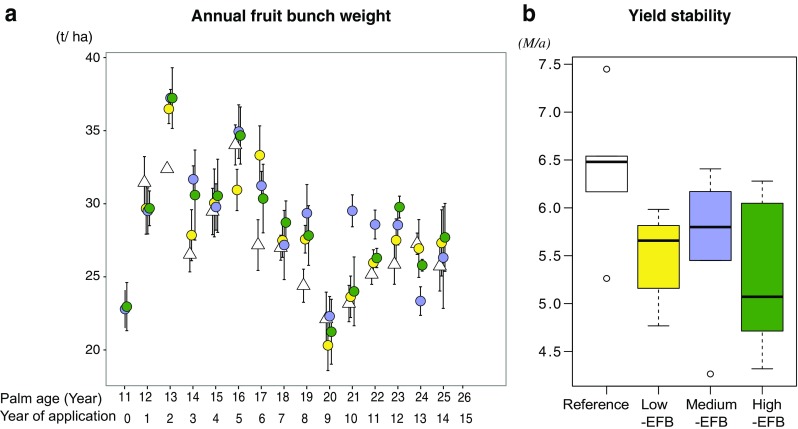
**a** Annual fruit bunch weight (mean ± SE, *n* = 5) over 15 years of application under four treatments: reference treatment (*open triangle*), low-EFB treatment (*yellow circle*), medium-EFB treatment (*blue circle*), and high-EFB treatment (*green circle*). Treatments did not significantly affect the annual crop yield (*F*_3202_ = 2.41, *P* = 0.068). **b** Inter-annual yield stability over 15 years under four treatments, represented as *boxplots* with median and upper and lower quartiles; the *whiskers* representing the maximum and minimum values; the *dots* are outliners. No significant differences in yield stability were detected among the four treatments (*F*_3,12_ = 2.35, *P* = 0.12). The units for yield stability is *μ*/*σ*, where *μ* is the temporal mean of crop yield and *σ* is the temporal standard deviation of crop yield over 15 years

We compared cumulative and annual crop yield between the four treatments. The cumulative crop yield of the five replicate plots over 15 years was 2040 t ha^−1^ under the chemical fertilizer treatment (Table 1). The cumulative crop yield under the low-EFB, medium-EFB, and high-EFB treatments increased by 2.4, 5.9, and 4.8%, respectively. The annual crop yield did not significantly differ between the treatments (*F*_3202_ = 2.41, *P* = 0.068) (Fig. 2a). These results suggest that switching from full chemical fertilizer treatment to the use of EFB as an alternative nutrient source led to slightly higher cumulative yield and similar levels of annual crop yield. The application rate of the high-EFB treatment (90 t ha^−1^ year^−1^) was higher than the business-as-usual practice of the low-EFB treatment (30 t ha^−1^ year^−1^) and medium-EFB treatment (60 t ha^−1^ year^−1^). However, the crop yield was not higher under the high-EFB treatment compared to that under the low-EFB treatment and medium-EFB treatment. This finding indicates that the current practice of EFB application is optimal for maintaining crop yield as chemical fertilizer-treated oil palm. A previous study in a Malaysian oil palm plantation showed that EFB application at the rate of 44 t ha^−1^ year^−1^ for 10 years resulted in a higher yield than chemical fertilizer treatment, while a lower rate of 22 t ha^−1^ year^−1^ of EFB application resulted in similar yield to the chemical fertilizer treatment (Abu Bakar et al. 2010). Advancing from the study, our findings further demonstrate that oil palm yield reaches a plateau with EFB application of an optimal rate.

We observed a pronounced temporal change in annual crop yield over 15 years (Fig. 2a). Specifically, the annual crop yield decreased from a palm age of 16 years and reached the lowest production at the age of 20 years. We hypothesized that EFB treatments may lead to lower temporal stability compared to chemical fertilizer treatment, because the amounts of nutrients released from EFB each year may vary due to variations in climatic conditions and field implementation. We found that the inter-annual yield stability under EFB treatments of all application rates appeared to be lower compared to that under the chemical fertilizer treatment; however, the differences were not statistically significant (*F*_3,12_ = 2.35, *P* = 0.12) (Fig. 2b). This result suggests that temporal stability in crop production at our study site was not strongly influenced by switching from chemical fertilizer treatment to EFB applications.

### Influence of climatic factors on temporal stability of production

**Fig. 3:**
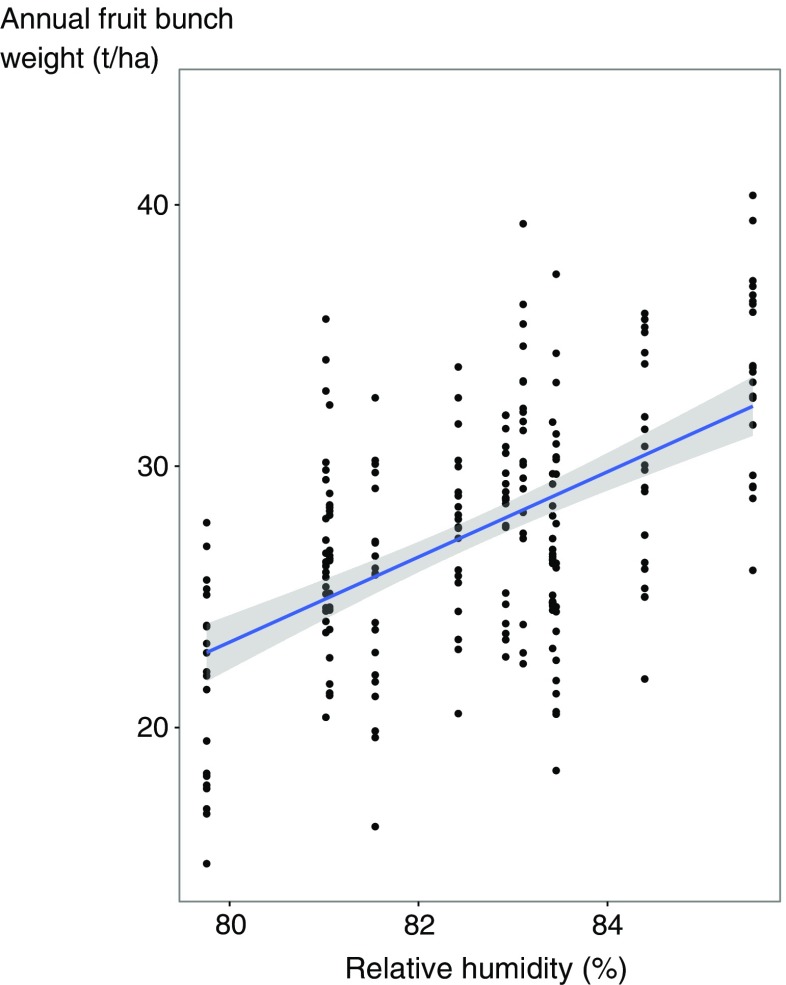
Annual fresh fruit bunch weight in a function of relative humidity with a lag effect of 2 years (*F*_1,9_ = 25.84, *P* < 0.001, *R* ^2^ = 0.30)

The temporal stability of crop yield was strongly influenced by climatic conditions. We observed that relative humidity significantly and positively influenced crop yield with a lag effect of 2 years (*F*_1,9_ = 25.8, *P* < 0.001) (Fig. 3). Specifically, during the yield decline period, of palm ages from 16 to 20 years the relative humidity decreased from 84 to 79%, the minimum temperature dropped from 22.5 to 17.4 °C, and the annual rainfall decreased from 2773 to 1955 mm year^−1^. This indicates a cooler environment with potential soil water deficiency, was sub-optimal for oil palm growth and fruit bunch production (Goh 2000). Similarly, it has been reported that seasonal changes in rainfall explain 55% of yield variations in Malaysian oil palm plantations (Chow 1992), while inter-annual variations in temperature and rainfall due to El Niño strongly influenced oil palm yield (Cadena et al. 2006). Our findings suggest that the effects of climatic conditions on oil palm yield may be more pronounced than the effects of crop residue management. Stronger effects of climatic conditions on yield over crop residue treatment have also been observed in various annual cropping systems (Marinari et al. 2015; Rusinamhodzi et al. 2011; Ventrella et al. 2016).

### EFB application influences crop yield by increasing soil organic carbon

**Fig. 4:**
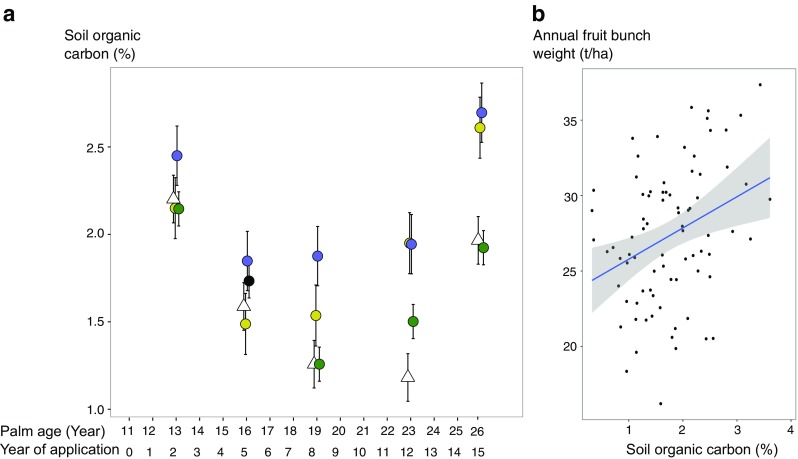
**a** Changes in soil organic carbon (mean ± SE, *n* = 5) over 15 years under four treatments: reference treatment (*open triangle*), low-EFB treatment (*yellow circle*), medium-EFB treatment (*blue circle*), high-EFB treatment (*green circle*), and reference treatment (*open triangle*). Soil organic carbon was significantly different between the treatments (*F*_3,92_ = 2.9, *P* < 0.05). **b** Annual fresh fruit bunch weight as a function of soil organic carbon, with a time lag of 2 years (*F*_1,78_ = 8.4, *P* < 0.05, *R* ^2^ = 0.11)

We observed increases in cumulative crop yield under EFB treatment of all application rates, compared to the chemical fertilizer treatment (Table 1). To understand whether the marginal positive effects of EFB treatments on crop yield were associated with soil organic carbon level, we firstly tested if soil organic carbon differed with treatment type (H3.1), and if soil organic carbon levels influenced crop yield (H3.2). We found that soil organic carbon at 0–15-cm depth significantly differed among the treatment types over the application period (*F*_3,92_ = 2.9, *P* < 0.05) (Fig. 4a). Specifically, the post hoc comparisons showed that soil organic carbon was significantly higher under the medium-EFB treatment (2.16 ± 0.17%; mean ± SE), compared to that under the reference treatment (1.64 ± 0.14%). Furthermore, soil organic carbon positively explained the annual crop yield with lag effects of 2 years (*F*_1,78_ = 8.4, *P* < 0.05, *R* ^2^ = 0.11) (Fig. 4b). As both soil organic carbon and the cumulative crop yield were the highest under the medium-EFB treatment among all the treatments, these results suggest that EFB application may increase cumulative crop yield by increasing soil organic carbon, especially under the medium-EFB treatment. EFB addition to the soil may increase soil organic carbon by decomposition and improved nutrient recovery (Baharuddin et al. 2009; Thambirajah et al. 1995). Increases in soil organic matter are associated with increased porosity, aggregate stability, hydraulic conductivity, and biological activities, which facilitate nutrient cycling and crop production (Edmeades 2003; Magdoff and Weil 2004). The positive effects of soil organic carbon on crop yield have been reported for annual crops, such as wheat, rice, maize, and peas (Lal 2010). Our findings present valuable empirical evidence for a tropical perennial cropping system.

## Conclusion

The land area under oil palm cultivation is expected to expand over the coming decades, not only in Southeast Asia but also in Africa and South America (Sayer et al. 2012). Identifying and implementing optimal management practices that can conserve soil ecosystem while intensifying crop yield is essential for the sustainable development of oil palm. Our study addressed a critical research gap of the effects of crop residue management on crop yield and its temporal stability, with a focus on comparing chemical fertilizer treatment with the application of empty fruit bunch (EFB). Results from a long-term trial at an Indonesian oil palm plantation showed that cumulative crop yield over 15 years was increased under EFB treatments compared to that under the chemical fertilizer treatment. The annual crop yield and temporal stability were not significantly different between the treatments. Crop yield was positively associated with relative humidity and soil organic carbon with a time lag of 2 years. These results convey an important message that switching from chemical fertilizer treatment to crop residue application maintained crop yield and temporal stability of production. Our findings also reinforce the importance of returning crop residues to agricultural fields for increasing soil carbon and sustaining soil fertility (Liska et al. 2014). Furthermore, climatic conditions over time had strong effects on the temporal variability of oil palm yield. With pressure for more sustainable practices within the oil palm industry and changing climatic conditions, optimizing agricultural management practices to maintain soil health will become even more important if intensification of oil palm is to expand in a sustainable manner. We have taken a step in this direction by highlighting that crop residue application maintained crop yield and temporal stability of oil palm production.
